# Decision Dynamics in Early Numerical Estimation: Evidence from the Dual-NLET and Drift Diffusion Modeling

**DOI:** 10.3390/jintelligence14030035

**Published:** 2026-02-25

**Authors:** Maybí Morell-Ruiz, Eva-Maria Ternblad, Betty Tärning, Sonja Holmer, Magnus Haake, Agneta Gulz

**Affiliations:** Department of Philosophy and Cognitive Science, Lund University Cognitive Science, Helgonavägen 3, 223 62 Lund, Sweden; eva-maria.ternblad@lucs.lu.se (E.-M.T.); betty.tarning@lucs.lu.se (B.T.); sonja.holmer@lucs.lu.se (S.H.); magnus.haake@lucs.lu.se (M.H.); agneta.gulz@lucs.lu.se (A.G.)

**Keywords:** Number-Line Estimation, Approximate Number System, Symbolic Number System, developmental numerical cognition, diffusion modeling, evidence accumulation paradigm, decision-making

## Abstract

The present study examined the cognitive mechanisms underlying decision-making in number-line estimation in 26 preschoolers through the lens of the evidence-accumulation paradigm. Children completed a traditional Number Line Estimation Task (NLET) and the Numeracy Screener test, which assessed symbolic and nonsymbolic abilities. They also completed a novel two-alternative forced-choice version of the Number Line Estimation Task (dual-NLET), which is introduced in this study as a tool for investigating decision-making processes in number-line estimation by enabling two-choice diffusion modeling. Results showed that accuracy in the traditional NLET correlated with both accuracy and decision efficiency in the dual task. Moreover, symbolic and nonsymbolic numerical abilities were differentially associated with distinct decision-making aspects: symbolic skills correlated with decision efficiency, while nonsymbolic skills correlated with decision threshold. These findings provide new insights into the roles of symbolic and nonsymbolic numerical systems in number-line decision-making and support the utility of the evidence-accumulation approach in developmental numerical cognition research.

## 1. Introduction

Numerical understanding is crucial not only for academic achievement but also for everyday decision-making. In children, foundational numerical systems emerge early and interact in complex ways as formal education begins. Understanding how these foundational systems develop and integrate is essential to improving mathematical learning outcomes.

Research in numerical cognition has identified two foundational systems that sup-port early mathematical thinking: the Approximate Number System (ANS) and the Symbolic Number System (SNS) (Knops, 2019). Despite their similarity, these systems follow different developmental paths and support different types of numerical representations.

The ANS enables intuitive, nonsymbolic quantity discrimination and emerges early in development (Feigenson et al., 2004; Starkey et al., 1990). It provides human—as well as primates—with a number sense for approximate numerical information. It is typically assessed using dot comparison tasks, where participants judge which of two dot arrays is more numerous (e.g., 7 vs. 9 dots). These tasks discourage counting and encourage fast, intuitive responses, such as assessed in the Numeracy Screener test (Nosworthy et al., 2013). Nonsymbolic abilities, as supported by the ANS, have been linked to a range of mathematical abilities, including arithmetic and estimation (Fazio et al., 2014; Schneider et al., 2017), suggesting that the ANS plays a foundational role in early numerical cognition.

In contrast, the SNS is acquired through formal education and cultural exposure. It allows children to manipulate exact quantities and understand concepts like numerical order and the distance between numbers. For example, recognizing that the difference between 6 and 7 is the same as between 186 and 187 depends on symbolic number knowledge. Symbolic abilities, as supported by the SNS, are commonly measured through symbolic comparison tasks, such as identifying the larger of two digits (e.g., 7 vs. 9), and are strongly related to broader math performance (Lyons et al., 2018).

While symbolic and nonsymbolic abilities have been studied extensively, the interrelation between ANS and SNS remains a matter of ongoing debate (Knops, 2019). The most prominent view—the mapping account—proposes that symbolic numbers gain meaning by being linked to existing nonsymbolic representations (Amalric & Dehaene, 2016; Dehaene & Cohen, 2007). Supporting evidence comes from correlational studies (Fazio et al., 2014), training interventions (Hyde et al., 2014; Park & Brannon, 2013), and neurocognitive findings (Piazza, 2010). However, alternative accounts suggest the relation may be bidirectional or even reversed, that is, symbolic knowledge might refine or reshape nonsymbolic representations, as suggested by the refinement account (Matejko & Ansari, 2016; Lyons et al., 2018; Lau et al., 2021). A key question is whether nonsymbolic representations fade as the symbolic system develops, or whether both systems remain functionally active and play complementary roles.

One promising way to investigate the integration between the ANS and the SNS is through the Number Line Estimation Task (NLET; Siegler & Opfer, 2003; Gunderson & Hildebrand, 2021, Wong et al., 2016; Morell-Ruiz et al., 2025), where children estimate the position of a symbolic number on a line marked only with two endpoints (e.g., from 0 to 10) and decide where the given target number should be located. In this study, we investigate how symbolic and nonsymbolic abilities interact when participants decide to mark a specific location on the NLET, with the aim of shedding light on the debate over whether numerical symbolic and approximative systems play complementary roles in numerical decision-making.

### 1.1. The Number-Line Estimation Task and Numerical Processing Abilities

In a 2003 study, Siegler and Opfer introduced the Number Line Estimation Task (NLET) as a tool to investigate the development of mental representations of numbers (Siegler & Opfer, 2003). In that study, the authors assessed the estimation skills of 96 children and 32 adults using the NLET. They found that the mental representation of numbers develops substantially with age, reflected in a shift from a nonlinear to a linear response pattern. Younger participants typically showed a logarithmic pattern, placing small numbers farther apart than expected and large numbers closer together than expected. By contrast, older children and adults showed a more accurate, linear pattern of estimation, in which consecutive numbers are spaced at roughly equal intervals. These estimation patterns were later confirmed in subsequent studies (Siegler & Booth, 2004; Opfer & Siegler, 2007), leading to the formulation of the log-to-linear shift theory.

To illustrate the difference between estimation patterns, imagine asking children to place the number 4 on a number line from 0 to 10. Younger children will tend to locate the target closer to, for example, number 7, reflecting a nonlinear, logarithmic pattern of estimation. Older children, in contrast, will place 4 near its correct position, showing a linear pattern. Importantly, the development of these estimation patterns depends on the numerical range (e.g., 0–10, 0–100, or 0–1000), with shorter ranges developing earlier. This means that the same older children who show a linear estimation pattern on the 0–10 number line in this example may still display a logarithmic pattern when estimating numbers in a larger range, such as 0–100.

Siegler and colleagues (Siegler & Opfer, 2003; Siegler & Booth, 2004; Opfer & Siegler, 2007) interpreted estimation patterns in the NLET as an index of the Mental Number Line (MNL). The idea that the cognitive system constructs an internal number representation organized from left to right was introduced by Stanislas Dehaene in his book Number Sense (Dehaene, 2011). According to the log-to-linear shift theory (see above), estimation patterns in the NLET can be viewed as an externalization of the MNL, providing researchers with a window into the developmental trajectory of the internal representation of numbers (Siegler & Booth, 2004; Opfer & Siegler, 2007).

The NLET as an index of the development of numerical estimation has received support from many studies reporting that accuracy in the NLET predicts high performance on math problems, such as arithmetic tasks, counting or standardized test for assessing early math abilities (Ashcraft & Moore, 2012; Bailey et al., 2014; Jordan et al., 2013; Hornung et al., 2014). Furthermore, in a meta-analysis by Schneider et al. (2018), the authors reported that the task is a robust tool for diagnosing and predicting math competencies. Taken together, these results indicate a strong link between symbolic abilities and accuracy in the NLET.

Regarding the relation between ANS acuity and accuracy in the NLET, the log-to-linear shift theory does not address what happens to nonsymbolic abilities or how they transform into symbolic representation of numbers, but some answers can be found in the mapping account mentioned above, which provides theoretical support for Siegler’s theory. The mapping account suggests that the ANS serves as the foundation for the later development of the SNS.

Building upon this approach that connects the theoretical proposal that the ANS is a foundation for the evolution of the SNS with the developmental perspective of the log-to-linear shift theory that suggests the NLET as a window for investigating the development of symbolic representations of number, some studies have investigated how the NLET can be used to enhance symbolic abilities in children, treating it as a bridge between the ANS and the SNS. Wong et al. (2016) found that NLET accuracy fully mediated the longitudinal relation between children’s ANS acuity and their symbolic arithmetic skills, suggesting that the task stimulates the key mechanism through which nonsymbolic representations support symbolic numerical development. In a recent intervention study, Morell-Ruiz et al. (2025) found that training with the traditional NLET strengthened the developmental pathway from nonsymbolic to symbolic magnitude processing in preschoolers, indicating that structured number-line practice can actively influence how the ANS supports the development of emerging symbolic abilities.

Taken together, the results from longitudinal and training studies reinforce the idea that the NLET can serve as a functional link between nonsymbolic and symbolic processing, aligning with the log-to-linear shift theory and the mapping account. However, these lines of research do not describe how the transition from ANS to SNS occurs. For example, what happens to the ANS during the process when the subject is deciding where to place the target number? How are the symbolic and nonsymbolic abilities playing complementary roles in NLET decision-making? While traditional NLETs provide insight into the mental representation of numbers—such as through development from log to linear estimation patterns—they offer limited information about the internal decision-making dynamics that unfold during estimation (see Ashcraft & Moore, 2012 for an insightful analysis of the cognitive mechanisms underlying number-line estimation).

Importantly, the log-to-liner shift theory has been subject to significant critique from the advocates of a proportional account (Barth & Paladino, 2011), although this account does not address the role of the ANS and the SNS in the NLET decision-making. The advocates of a proportional account argue that estimation in the NLET relies on underlying cognitive proportional abilities rather than an index of the MNL, questioning whether the link between accuracy in the NLET and the SNS abilities is an artefact, thus suggesting that children’s proportional abilities better predict the SNS abilities. Interestingly, while proportional abilities are assumed to be related to the ANS, the estimation pattern in the NLET is not seen as a product of MNL’s development from log to linear patterns; instead, the numerical cognitive system has only one basic representation, a power function.

A recent study by Gunderson and Hildebrand (2021) offers relevant developmental evidence for understanding number-line estimation. Using a robust longitudinal design with 612 children assessed across four occasions over two years, the authors examined how number-line estimation relates to a wide range of spatial and numerical skills. Their findings showed that number-line estimation was significantly associated with both exact and approximate symbolic calculation skills, even after controlling for proportional reasoning and other spatial measures. Although their study does not address the integration of symbolic and nonsymbolic systems, it highlights the central role of number-line estimation within children’s broader numerical development and underscores the need to investigate the cognitive mechanisms that support estimation beyond outcome-level performance.

Ashcraft and Moore (2012) addressed the need to investigate the cognitive mechanisms underlying number-line estimation by analyzing both accuracy and response time in children from grades 1 to 5. Their findings supported the log-to-linear shift theory, revealing developmental changes in estimation patterns and stronger associations with mathematical achievement across grade levels. However, while their study introduced separate analyses of response time and accuracy to explore the cognitive mechanisms involved in the NLET, it did not integrate these measures into a unified process-level analysis of the decision dynamics, nor did it examine how symbolic and nonsymbolic numerical systems contribute to number-line estimation.

In sum, there is a need for further investigation into the specific role that numerical abilities—symbolic and nonsymbolic—play in NLET decision-making. In our study, we investigate the specific contribution of each ability to the decision-making process in-volved in the participant’s choice of a location on the number line. To this end, we propose a parallel between estimation and decision-making, drawing on theories of decision-making based on evidence accumulation. In the following section, we describe this theoretical framework and explain how we apply it to the study of number line estimation.

### 1.2. Investigating the Cognitive Mechanisms Underlying Decision-Making Through the Lens of Evidence Accumulation Paradigm

Decision making has been studied in cognitive science as the process by which agents select one option among competing alternatives under conditions of uncertainty and time constraints (Fischhoff & Broomell, 2020). A well-established framework for analyzing such processes is the evidence accumulation paradigm, which conceptualizes decisions as the gradual integration of noisy information over time until a response threshold is reached (Stewart et al., 2006). Within this framework, momentary samples of evidence supporting different options are continuously evaluated and combined, allowing decisions to emerge dynamically rather than being determined by a single instantaneous judgment.

To illustrate this idea, consider a simple everyday decision such as choosing between two checkout lines at a supermarket. The decision maker does not have direct access to the true waiting time of each line, but instead relies on multiple imperfect cues, such as the number of people waiting, the apparent speed of the cashier, or prior experience with similar situations. Each cue provides noisy evidence favoring one alternative (e.g., line A) over the other (e.g., line B). As this evidence is sampled and accumulated over time, the decisionmaker gradually moves closer to committing to one choice. A decision is made once the accumulated evidence in favor of one alternative exceeds a sufficient threshold, reflecting a balance between selecting the better option and response speed.

The evidence accumulation paradigm formalizes this intuition by treating decision making as a stochastic process that jointly accounts for choice accuracy and response times, commonly implemented through two-choice diffusion modeling approaches (Ratcliff & McKoon, 2008). Among these, the Drift Diffusion Model (DDM) has become a central tool for studying binary decision making (Ratcliff & McKoon, 2008; Ratcliff et al., 2010; Ratcliff et al., 2012). In the DDM, evidence is modeled as a noisy accumulation process that evolves over time toward one of two response boundaries, capturing the trade-off between speed and accuracy. This process is characterized by a small set of interpretable parameters: drift rate, boundary separation, non-decision time, and starting point. The drift rate reflects decision efficiency of evidence accumulation; in the supermarket example, it captures how clearly the available cues favor one line over the other. Each boundary represents one of the two alternatives, and boundary separation represents the decision threshold (the amount of evidence required before committing to a decision), with wider boundaries indicating greater response caution and narrower boundaries reflecting a more confident decision. Together, these parameters describe the core accumulation process. In contrast, non-decision time accounts for processes outside the accumulation itself, such as sensory encoding and motor execution, while the starting point represents the initial state of the accumulation process and captures potential biases toward one alternative prior to evidence integration. For instance, an initial preference for a particular line (A or B) would be reflected in a starting point closer to the corresponding boundary. This framework enables researchers to decompose observed behaviors into underlying cognitive mechanisms, providing a detailed account of how decisions unfold over time rather than relying solely on aggregate measures like mean response times or accuracy.

Two-choice diffusion models have been widely applied to investigate the cognitive mechanisms underlying performance across diverse tasks, including perceptual discrimination, lexical decision making, memory, attention, and reasoning (Forstmann et al., 2016). A comprehensive review by Forstmann et al. (2016) offers an accessible overview of the advantages and applications of the Drift Diffusion Model (DDM) across cognitive domains, highlighting its usefulness for disentangling latent components of decision making. For instance, Ratcliff et al. (2010) showed that in tasks such as numerosity discrimination and lexical decision, decision efficiency (as measured by drift rate) varied with individual differences in IQ, whereas decision threshold (as measured by boundary separation) reflected strategic adjustments in caution. Similarly, White et al. (2010) used the DDM to reveal enhanced processing of threat-related words in anxious individuals, a pattern that traditional response time or accuracy measures failed to detect. In developmental contexts, Ratcliff et al. (2012) found that children exhibited slower sensory and motor processing (as reflected in longer non-decision times) and adopted higher decision thresholds (as indicated by wider boundary separation) than adults, consistent with increased response caution and slower perceptual–motor dynamics. Together, these findings suggest that two-choice diffusion modeling grounded in the evidence accumulation paradigm not only captures latent cognitive dynamics but also clarifies the distinct roles of underlying cognitive processes that remain obscured when relying solely on conventional analyses of accuracy or mean response times.

Building on this, Park and Starns (2015) applied two-choice diffusion modeling to numerical cognition tasks in 120 adult participants who completed a dot comparison task, reporting that higher drift rates were associated with high performance in nonsymbolic magnitude comparison tasks among university students (Park & Starns, 2015). Szardenings et al. (2018) examined children aged 8–10, both with and without dyscalculia, and showed that diffusion modeling effectively captured differences in the quality of numerical representations, as indicated by the correlation between drift rates and math task performance (Szardenings et al., 2018). In 2022, Spliethoff and colleagues applied the EZ-diffusion model to analyze performance in a dot comparison task with monetary incentives in a sample of twenty-three 7-year-old and thirty 14-year-old children and found that both age groups exhibited higher decision efficiency (as indexed by the drift rates) in a reward compared to a neutral condition (Spliethoff et al., 2022). Although these studies do not include the NLET and substantially differ in their research purposes, their findings indicate that two-choice diffusion models can reveal how adults and children accumulate evidence and reach decisions in numerical tasks.

Beyond two-choice models, Ratcliff and McKoon (2020, 2024) used an alternative approach called the Spatially Continuous Diffusion Model (SCDM; Ratcliff, 2018) to investigate NLET performance in 16 adults. In 2024, the authors used the SCDM to examine aging effects in numeracy tasks, including the NLET, comparing the performance among three groups of adults (young adults, 60–69, and 70–90 years old). On this occasion, they used an arc-shaped line and found differences between age groups in the NLET regarding non-decision time, indicating that aging is associated with longer durations of sensory encoding and motor execution before decision-making begins on the number line. However, the authors explicitly state that the SCDM should not be applied to children’s performance in the NLET until it has been automated: “The number-line task has been used extensively in the developmental domain. However, it is important not to apply the SCDM to data from children who have not yet automated the decision process … (i.e., when they have a linear representation)” (Ratcliff & McKoon, 2020, p. 21). This constraint limits the application of this alternative diffusion model for the investigation of the development of number-line abilities.

Overall, to our knowledge, no study has applied two-choice diffusion models to examine dynamic decision-making in the context of the NLET. Furthermore, no research has employed diffusion models—including the two-choice model and the SCDM—to investigate children’s performance in this task, or to explore how the ANS and SNS contribute to this process. For instance, which numerical system, nonsymbolic or symbolic, is primarily associated with decision efficiency (drift rates) in the NLET? Are either of them related to the decision threshold (boundary separation)?

To address this gap, the present study applies the evidence accumulation paradigm to investigate the dynamics of ANS–SNS integration during number line estimation. To make this analysis possible, we developed a novel version of the NLET that transforms the estimation process into a binary decision task suitable for modeling with the two-choice diffusion models.

### 1.3. A Dual-NLET to Support Evidence Accumulation Modeling

In the traditional NLET format introduced by Siegler and Opfer (2003), children are presented with a number line bounded by labeled endpoints (e.g., 0 and 10) and asked to locate a given target number on the line. Performance on the NLET has been shown to correlate robustly with broader mathematical competence across age groups and educational levels (see Schneider et al., 2018, for a meta-analysis). This association holds across multiple mathematical outcomes, including counting, arithmetic, and standardized achievement tests, and remains significant even after controlling for variables such as working memory, intelligence, and socioeconomic status (Bailey et al., 2014; Jordan et al., 2013; Hornung et al., 2014).

The predictive power of the NLET appears stable across task formats with or without a mark in the line (Siegler & Opfer, 2003; Schneider et al., 2018). While the traditional version follows a number-to-position (NP) format, the alternative position-to-number (PN) variant, suggested by Siegler and Opfer (2003), encourages children to estimate the number corresponding to a mark along the line. Evidence suggests that both formats engage similar underlying mechanisms. Siegler and Opfer (2003), for instance, found that NP and PN tasks elicit comparable developmental patterns, with children transitioning from logarithmic to linear mappings in both cases. Moreover, Schneider et al. (2018) report that the PN variant—though less frequently used—shows a similar level of correlation with mathematical competence as the NP format, reinforcing the view that both tasks tap into a shared magnitude representation system. Both the NP and PN format, however, share a key characteristic: they require open-ended responses—either a position on the line or a numerical estimate—thereby limiting the applicability of diffusion models based on binary decisions.

To apply the evidence accumulation paradigm to the NLET using the two-choice diffusion model, we developed a new dual-format NLET version of the task, inspired by the PN format but adapted into a two-alternative forced-choice (2AFC) design. In this dual-NLET, each trial presents a target number along with two marked positions on the number line: one representing the correct location (the target mark) and the other a distractor (the distractor mark) separated by ±1 unit (e.g., if the target is 7, the distractor mark might be placed at 6 or 8). Children are asked to select which of the two marks best corresponds to the target number. This design requires children to map a symbolic number onto a spatial position on the number line (as in the traditional NLET) and to resolve competition between two nearby candidate locations, thereby framing number-line estimation as a decision between two competing alternatives: the target and the distractor marks. Although the response format differs from the traditional NLET, both tasks rely on the same core mapping operation from numerical representations to spatial location; accordingly, individual differences in traditional estimation accuracy are expected to relate to both behavioral performance and decision efficiency in the dual-NLET.

The dual-NLET—by converting estimation into a binary-choice format—opens the possibility of applying the two-choice diffusion models to the number-line estimation process, allowing a dynamic investigation of the process and the study of integration between symbolic and nonsymbolic abilities at the process level. This novel format allows us to explore, for example, whether symbolic knowledge primarily influences the decision efficiency of evidence or whether nonsymbolic intuition shapes the decision threshold that children adopt when faced with uncertainty. In doing so, we aim to uncover how foundational numerical systems interact during number-line estimation and how they contribute to individual differences in numerical decision-making. By adapting the task format (dual-NLET) and integrating process-level analysis using two-choice diffusion modeling, our study introduces a novel approach to investigating the integration of numerical systems during number reasoning.

### 1.4. Purpose and Hypotheses

This study introduces the dual-NLET—a two-choice version of the number-line estimation task—designed to investigate children’s numerical decision-making from a dynamic perspective. Our primary objectives are to test whether this task enables (1) the study of number-line estimation from a dynamic perspective, and (2) the investigation of the integration of symbolic and nonsymbolic abilities through the lens of evidence accumulation.

Specifically, we hypothesize that (H1) *accuracy in the traditional NLET will correlate with accuracy in the dual-NLET*, indicating a common underlying ability across formats. We also expect that (H2) *accuracy in the traditional NLET will correlate with decision efficiency in the dual-NLET, as measured by drift rate parameter in diffusion modeling*. By linking number-line estimation skills in the traditional task (NLET) with decision efficiency in the dual-NLET, this study aims to investigate whether these tasks relate to each other at the outcome and process levels.

Finally, regarding the role of symbolic and nonsymbolic abilities in number-line estimation, building on previous literature, we anticipate that both abilities will be integrated into the decision-making process of the dual-NLET, but will be associated with different aspects of number-line dynamics. Specifically, we hypothesize that (H3) *symbolic abilities, as measured by the Numeracy Screener test, will be associated with decision efficiency in the dual-NLET, indexed by the drift rate parameter in diffusion modeling* (H3a)*, whereas nonsymbolic abilities, also measured by the Numeracy Screener test, will be associated with decision threshold, indexed by the boundary separation parameter in diffusion modeling* (H3b). By investigating the association between numerical abilities and number-line estimation using this approach, this study examines whether symbolic and nonsymbolic abilities play distinct roles in the number-line decision process, with the aim of providing evidence relevant to SNS–ANS interaction in numerical decision-making.

## 2. Materials and Methods

### 2.1. Participants and Ethical Considerations

The study was part of a broader research project on early mathematical competencies, involving six preschool classes, where one class was randomly selected for this study from the six involved in the larger project. No selection criteria were applied, resulting in a sample of 26 preschoolers (13 girls and 13 boys). At the start of the study, participants’ age ranged from 5;9 to 6;9. All children were enrolled in the same preschool located in the vicinity of Lund, Sweden, and came from medium to high socioeconomic backgrounds.

The study project was submitted for consideration to the Swedish Ethical Review Authority (2023-03043-01) and was conducted in accordance with ethical regulations for research involving children. Participation was voluntary; parents could opt out. Children were informed of their right to decline participation during an information session conducted in the classroom prior to data collection, with the presence and prior agreement of the classroom teacher.

### 2.2. Procedure

Data was collected across two sessions between December 2023 and January 2024. During the first session, children completed two tasks: the Numeracy Screener test (Nosworthy et al., 2013), assessing symbolic and nonsymbolic numerical abilities, and the traditional bounded Number Line Estimation Task (NLET; Siegler & Opfer, 2003). During the second session, number-line estimation was assessed again using the dual-NLET, a two-alternative forced choice version designed for this study.

Both sessions took place during the morning hours, in a quiet room within the school building. Children were tested individually by trained experimenters. The order of task administration in the first session was counterbalanced across participants to control order effects. The first session lasted approximately 10–15 min per child, while the second session was shorter, lasting about 5 min.

### 2.3. Measurements

Participants in the study completed three tasks: the Numeracy Screener test (Section 2.3.1), the traditional Number Line Estimation Task (Section 2.3.2) and the dual-NLET (Section 2.3.3).

#### 2.3.1. Numeracy Screener Test: Symbolic and Nonsymbolic Numerical Abilities

Children’s symbolic and nonsymbolic abilities were assessed using the *Numeracy Screener* test (Nosworthy et al., 2013), a paper-based instrument designed to evaluate symbolic and nonsymbolic magnitude processing. The task consisted of two blocks: a *Nonsymbolic Trials* (NST) block using dot arrays, and a *Symbolic Trials* (ST) block using Arabic numerals.

**Figure 1:**
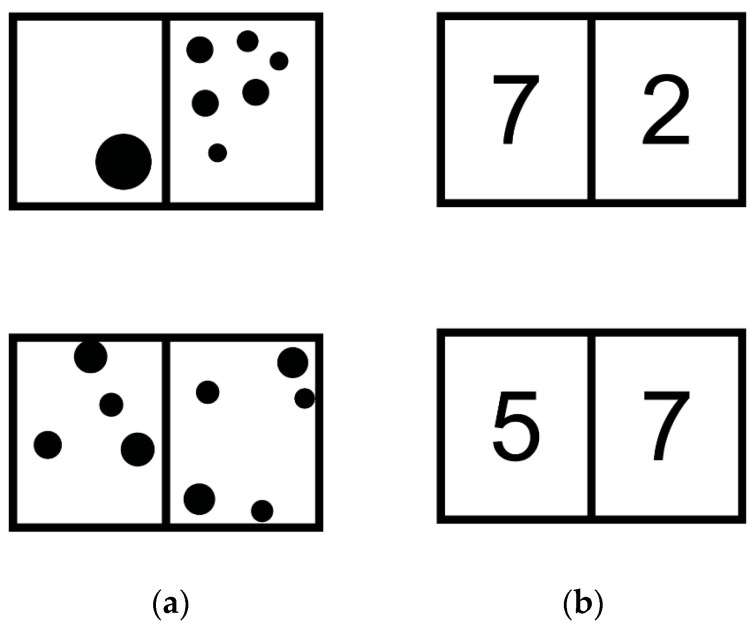
Examples of trials in the Numeracy Screener test: (**a**) Nonsymbolic Trials (NST); (**b**) Symbolic Trials (ST).

In the NST block, children were shown 56 pairs of dot arrays and asked to mark the array that contained more dots. Figure 1a illustrates two examples of the NST with 1 and 6 dots in the top trial, and 4 and 5 dots in the bottom trial. To provide their answers, participants had to draw a line with a pencil on top of the set with 6 dots in the top trial and do the same for the set with 5 dots in the bottom trial.

Similarly, in the ST block, they were shown 56 pairs of symbolic numerals and asked to indicate which number was larger. Figure 1b illustrates one example of ST on top (7 vs. 2) and another on the bottom (5 vs. 7), with the correct answer being 7 in both cases.

Each block was time-limited to one minute. Before starting each block, the experimenter explained the task and provided practice trials for the participant. Instructions were delivered orally by the experimenter, who also guided each child through a short practice session before beginning the timed trials.

Of the initial 26 participants, 2 did not complete the Numeracy Screener test: one was absent during the data collection session, and the other provided only a partial completion. Participants’ task accuracy was computed using adjusted symbolic and nonsymbolic scores following Lyons et al. (2018), which contrast correct and incorrect responses and scale this difference by the number of response alterna-tives. Because the task is binary (two response options), this scaling term equals 1, and the adjusted accuracy for-mula reduces to the difference between correct and incorrect responses. This yielded two adjusted accuracy scores per participant: one for NST and one for ST. Higher adjusted NST scores reflect greater nonsymbolic abilities, whereas higher adjusted ST scores reflect greater symbolic abilities. 

#### 2.3.2. Number Line Estimation Task (NLET): Traditional Format

**Figure 2:**
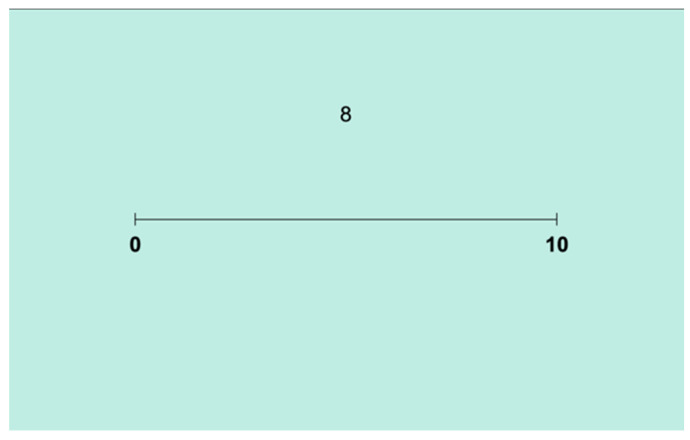
Trial example of a testing phase in the traditional Number Line Estimation Task (NLET). The child is asked to estimate the location of the number 8 (target number) on a number line bounded by 0 and 10. The response is given by tapping the screen at the perceived location.

Children’s number line estimation skills were assessed using the traditional version of the NLET (Siegler & Booth, 2004) in the first session. Figure 2 illustrates this task, where children are shown a horizontal number line labeled only at the endpoints (e.g., 0 and 10) and asked to estimate the location of a target number (e.g., 8).

Pre-recorded instructions were delivered in Swedish through the iPad audio system, while an experimenter remained present to support the child during practice trials, where target numbers 1, 2 and 3 where used. No feedback or intermediate markers were provided. The task was administered on an iPad, and children responded by touching the location on the screen where they believed the target number should be placed.

During testing, the list of targets included all integers from 1 to 9, presented in random order while controlling for repetitions. Each participant estimated one target number per trial, totaling 9 trials. No time limit was set for each trial. The session lasted approximately 5 min per participant.

Of the initial 26 participants, 3 did not complete the traditional NLET (one absent during data collection; two incomplete). Participants’ performance in the NLET was scored following the methodology outlined by the seminal study of Siegler and Booth (2004). Percent Absolute Error (PAE) was used as the primary metric to quantify estimation accuracy, calculated as PAE = 100 × (Estimate − Estimated Quantity)/(Scale of Estimate), where Estimate represents the value estimated by the child (indicated by the click location on the line), Estimated Quantity is the actual target number, and Scale of Estimate refers to the range of the number line (e.g., 10 for a line ranging from 0 to 10). Lower PAE values indicate greater accuracy in the traditional NLET.

#### 2.3.3. Dual Format of the Number Line Estimation Task: The Dual-NLET

During the second session, children completed a modified version of the number line task, referred to as the dual-NLET, developed specifically for this study. Like the traditional format, in each trial of the dual-NLET children were presented with a number line bounded by 0 and 10, along with a target number (e.g., 7). In the dual-NLET, in contrast, participants provide their answers by choosing between two marked positions on the line. One position corresponded to the correct location of the target (target mark), while the other served as a distractor (distractor mark) dislocated by one unit.

**Figure 3:**
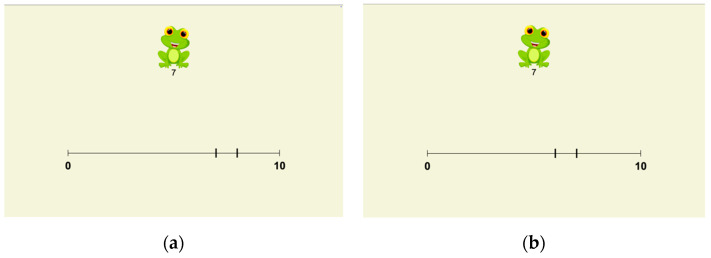
Example trials from the dual version of the Number Line Estimation Task (dual-NLET). In each trial, the child is asked to locate the number 7 by choosing between two marked positions on a 0–10 number line: (**a**) the distractor is placed to the right of the target (distractor mark right); (**b**) the distractor is placed to the left (distractor mark left).

Figure 3 illustrates two trials of the dual-NLET with the same target number, 7 in this case. Figure 3a shows the distractor mark to the right of the target mark (distractor mark right), while Figure 3b depicts the distractor mark to the left of the target mark (distractor mark left). Each target number from 1 to 9 was included in two trials—one alongside a distractor mark to the left and another alongside a distractor mark to the right—for a total of 18 trials.

The ±1-unit spacing between target and distractor was chosen for methodological reasons. First, this distance allows each target value (1–9) to appear in two symmetric conditions, with the distractor located either to the left or to the right of the target, ensuring balanced spatial presentation across trials. Second, given the bounded range of the number line (0–10), larger distractor distances would prevent several target values from supporting symmetric left–right pairings, which would either reduce the number of usable targets or require an unbalanced design. The ±1 spacing therefore represents the largest distance that preserves both symmetry and full coverage of target values, while maintaining a constant local difficulty across trials.

The task was administered on an iPad, and children responded by tapping the target mark or the distractor mark. Responses and reaction time were automatically stored. To increase engagement, a cartoon frog character appeared on the screen, introducing a short narrative in which the child was asked to help find the correct number. Instructions were delivered in Swedish using a child-friendly recorded voice.

Of the initial 26 participants, 2 did not complete the dual-NLET due to absence during the second data collection session. Choice responses were used to compute dual-NLET accuracy (percentage of correct responses) and were also included as inputs for the two-choice diffusion modeling, whereas reaction times were recorded and used only for modeling. A response was considered correct when the child selected the target mark, and incorrect when the child selected the distractor mark. Each correct response was assigned a score of 1, and each incorrect response a score of 0. For each participant, the number of correct responses out of 18 trials was divided by the total number of trials and multiplied by 100 to obtain individual accuracy percentages. Higher percentages of correct responses indicated higher accuracy in the dual-NLET.

### 2.4. Analysis

In our analysis, we investigated the relation between children’s abilities to process numerical information—measured through both symbolic and nonsymbolic skills—and their decision-making in number line estimation. To explore this relation, we examined children’s decision-making in the number line task using the evidence accumulation paradigm via the dual variant of the NLET. Our analysis consisted of two steps. First, we assessed whether the novel dual-NLET and the traditional NLET were related by examining the correlations between accuracy in the traditional NLET and accuracy (H1) and decision efficiency in the dual-NLET (H2). Then, we analyzed the relation between abilities to process numerical information and decision-making dynamics in the dual-NLET by examining (H3a) the correlation between symbolic numerical abilities and decision efficiency in the dual-NLET, as measured by the HDDM drift rate parameter, and (H3b) the correlation between nonsymbolic numerical abilities and decision threshold in the dual-NLET, as measured by the HDDM boundary separation parameter.

Altogether, there were three groups of variables included in the analysis: (1) accuracy in both the traditional NLET (as measured by the Percentage of Absolute Error) and the dual-NLET (as measured by the percentage of correct responses); (2) decision-making dynamics in the dual-NLET (as measured by decision efficiency and decision threshold); and (3) abilities to process numerical information (as measured by symbolic and nonsymbolic numerical skills).

Diffusion modeling was performed on a subset of participants (*N* = 22) who completed the dual-NLET and provided valid response time data. Of the initial sample (*N* = 26), two participants did not complete the dual-NLET due to absence during the second data collection session, and two additional participants were excluded from the HDDM analysis due to excessively long response times, resulting in a final subset of 22 participants (see Section [Diffusion Modeling] for a description of the diffusion modeling methodology).

From this subset of 22 participants, only 17 had complete data across all tasks (Numeracy Screener, traditional NLET, and dual-NLET) and were therefore included in the correlational analyses reported in the Results section. Specifically, two participants did not complete the Numeracy Screener test (one absent during data collection; one incomplete), and three did not complete the traditional NLET (one absent during data collection; two incomplete). A summary of the measurements for this final subset of 17 participants is provided in Table 1.

Since we were analyzing correlations between variables, we first checked whether each variable followed a normal distribution using the Shapiro–Wilk test (see Table 1). When both variables were normally distributed, Pearson’s r was used to analyze correlations; otherwise, Spearman’s *ρ* was applied. For effect-size interpretation, Pearson’s *r* was evaluated according to Cohen’s (1988) benchmarks, whereas Spearman’s *ρ* was interpreted following the nonparametric guidelines proposed by Dancey and Reidy (2007).

To evaluate H1, we examined the correlation between accuracy in the traditional NLET, measured by the Percentage of Absolute Error (PAE), and accuracy in the dual-NLET, measured by the percentage of correct responses. To evaluate H2, we analyzed the correlation between accuracy in the traditional NLET, measured by the Percentage of Absolute Error (PAE), and decision efficiency in the dual-NLET, measured by the drift rate. Because normality assumptions were violated for at least one variable in each comparison, Spearman’s rank correlation was used for both analyses.

Finally, we tested H3 in two parts. First, we examined the relation between symbolic abilities and decision efficiency in the dual-NLET, as measured by drift rate (H3a). Second, we analyzed the relation between nonsymbolic abilities and decision threshold in the dual-NLET, as measured by boundary separation (H3b). Because normality assumptions were met for all variables involved in H3, Pearson’s correlation was used.

#### Diffusion Modeling

We analyzed children’s decision-making in the dual-NLET using diffusion modeling, a dynamic, evidence-accumulation-based approach (Ratcliff & McKoon, 2008). To do so, we implemented the Hierarchical Drift Diffusion Model (HDDM), which is a Bayesian approach to the DDM (Wiecki et al., 2013). The HDDM estimates individual-level parameters as draws from group-level distributions, where each participant’s drift rate, boundary separation, starting point, and non-decision time are treated as random effects, with their hyperparameters inferred jointly at the population level (Wiecki et al., 2013). According to Wiecki et al. (2013), HDDM fixes the diffusion coefficient at 1 and provides informed default priors for all parameters, thereby avoiding the need for manual prior specification. The model employs Markov Chain Monte Carlo sampling to obtain posterior distributions of the latent decision components, producing hierarchical estimates that combine information across participants while preserving individual variability (Wiecki et al., 2013).

Before modeling, we applied a data-cleaning procedure to remove extreme response times that did not reflect the actual decision process of interest. Visual inspection of the response time distribution revealed a small number of exceptionally long responses, and trials exceeding 15,000 ms were treated as outliers. As these prolonged responses were concentrated on only two participants, their data was excluded from the diffusion modeling analysis.

Data modeling was conducted with 22 participants, as 2 were excluded due to extremely long response times and 2 did not complete the task (one absent during data collection; one incomplete). Participants’ individual scores of reaction time and accuracy in every trial of the dual-NLET were both considered inputs for the model. For the remaining participants (*N* = 22), the average correct response time was 3.76 s (*SD* = 1.93), while the average incorrect response time was 3.69 s (*SD* = 1.91).

The model was executed for approximately 1 h and 10 min, using 50,000 samples and a burn-in period of 30,000 iterations. The model provides participant level estimates for four parameters describing distinct components of the decision process in the dual NLET. Decision efficiency (drift rate) reflects how efficiently participants resolve the competition between the target and distractor marks, that is, how rapidly they tend, on average, to commit to the target mark relative to the distractor (*M* = 0.34; *SD* = 0.05). Decision threshold (boundary separation) indexes the amount of integrated information required before committing to either the target or the distractor mark, regardless of response correctness (*M* = 2.90; *SD* = 0.51). Starting point captures initial bias toward one of the two marks, reflecting the tendency to begin the decision process closer to either alternative (target or distractor) prior to information integration (*M* = −0.11; *SD* = 0.007). Finally, non-decision time reflects processing components unrelated to the choice itself, such as stimulus encoding and motor execution, capturing delays that occur outside the core decision process (*M* = 1.55; *SD* = 0.24).

These parameters were subsequently used in analyses to explore how children’s decision-making dynamics in the dual-NLET relate to accuracy in the traditional NLET and their numerical processing skills in symbolic and nonsymbolic trials in the Numeracy Screener test. In our study, we examined decision efficiency, measured by drift rate, and the decision threshold, measured by boundary separation, to analyze decision-making in the dual-NLET. We did not include the other two parameters—the HDDM starting point and non-decision time parameters—in our hypothesis testing, as they were not relevant to our main hypotheses.

## 3. Results

### 3.1. Accuracy in the Traditional NLET and the Dual-NLET

This section tests H1 by examining the relation between accuracy in the traditional NLET (PAE) and accuracy in the dual-NLET, as measured by the percentage of correct responses. Because at least one of the variables violated normality assumptions (see Table 1), associations were assessed using Spearman’s rank correlation.

The analysis revealed a significant negative correlation between the percentage of absolute errors in the traditional NLET and the percentage of correct responses in the dual-NLET (*N* = 17; Spearman’s *ρ* = –0.54, *p* = .02). This corresponds to a moderate correlation according to the guidelines proposed by Dancey and Reidy (2007).

**Figure 4:**
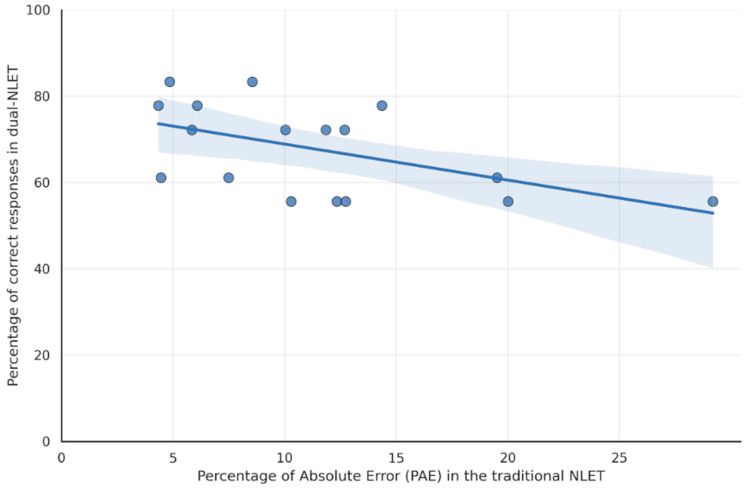
Pearson’s r correlation between the Percent Absolute Error (PAE) in the traditional Number Line Estimation Task (NLET) and the percentage of correct responses in the dual Number Line Estimation Task (dual-NLET).

Figure 4 illustrates this correlation, with traditional NLET accuracy on the x-axis and dual-NLET accuracy on the y-axis. As shown, lower estimation error in the traditional task was associated with higher accuracy in the dual task. These findings support H1, suggesting that children with stronger number-line estimation skills, as assessed by the traditional NLET, tend to perform better in the dual-choice estimation task. These results indicate a degree of stability in numerical estimation across modalities and support the dual task as a possible tool for further investigation of estimation abilities.

### 3.2. Accuracy in the Traditional NLET and Decision Efficiency in the Dual-NLET

This section tests H2 by examining the relation between accuracy in the traditional NLET (PAE) and decision efficiency in the dual-NLET, as measured by drift rate. Because normality assumptions were violated for one of the two variables (see Table 1), associations were assessed using Spearman’s rank correlation.

Results indicate a significant negative correlation between the variables (*N* = 17; Spearman’s *ρ* = –0.52, *p* = .03). This corresponds to a moderate correlation according to the guidelines proposed by Dancey and Reidy (2007).

**Figure 5:**
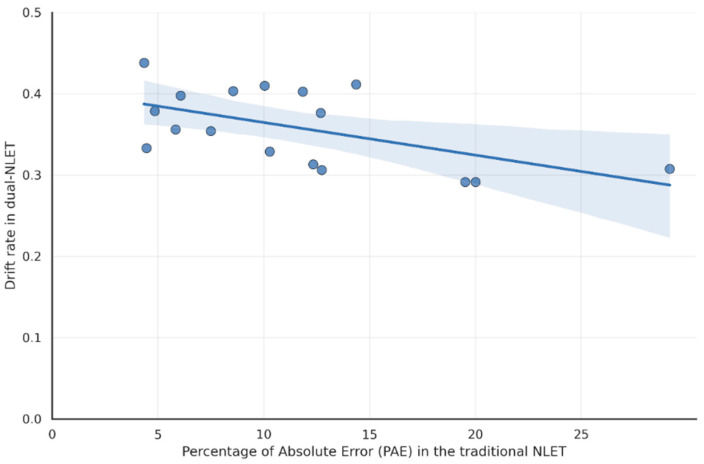
Pearson’s r correlation between the Percent Absolute Error (PAE) in the traditional Number Line Estimation Task (NLET) and drift rate in the dual Number Line Estimation Task (dual-NLET).

Figure 5 depicts this correlation, with traditional NLET accuracy (PAE) on the x-axis and dual-NLET drift rate on the y-axis, showing that a lower percentage of estimation errors was associated with a higher dual-NLET drift rate. These results support H2, indicating that children who performed more accurately on the traditional task tend to accumulate evidence more efficiently in the dual modality, reflecting consistency across task demands at both the outcome and process levels.

### 3.3. Symbolic and Nonsymbolic Numerical Abilities in the Decision-Making Process of the Dual-NLET

This section tests H3 by examining, in two parts, how symbolic and nonsymbolic numerical abilities relate to distinct components of decision-making dynamics in the dual-NLET (H3a and H3b). Because normality assumptions were met for all variables involved (see Table 1), associations were assessed using Pearson’s correlation.

First, addressing H3a, results indicated a significant positive correlation between symbolic abilities and decision efficiency (*r*(15) = 0.50, *p* = .04), suggesting that higher symbolic skills were associated with greater decision efficiency. According to Cohen’s (1988) guidelines, this corresponds to a large effect size.

Second, addressing H3b, the analysis showed a significant negative correlation between nonsymbolic abilities and decision threshold (*r*(15) = –0.51, *p* = .03), indicating that higher nonsymbolic skills were associated with lower decision threshold in the dual-NLET. According to Cohen’s guidelines, this also corresponds to a large effect size.

**Figure 6:**
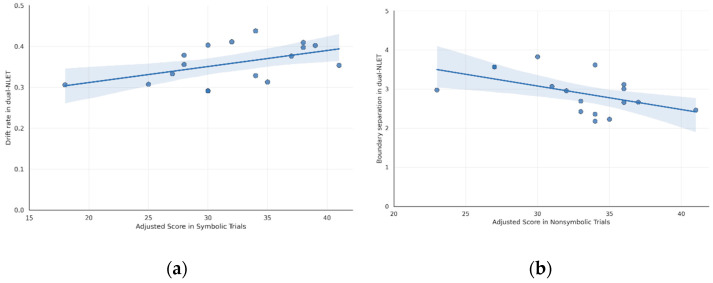
Pearson’s *r* correlations in the dual Number Line Estimation Task (dual-NLET): (**a**) correlation between symbolic abilities and drift rate; (**b**) correlation between nonsymbolic abilities and boundary separation.

These findings are illustrated in Figure 6, with the left panel showing results from the first part of the analysis (H3a) and the right panel showing results from the second part (H3b). The figure shows that children with higher symbolic skills exhibited higher decision efficiency in the dual-NLET, suggesting that this ability plays a key role in the number-line estimation process. On the other hand, children with higher nonsymbolic skills exhibited a lower decision threshold, indicating greater confidence in their decisions. Taken together, these findings support H3, indicating that symbolic and nonsymbolic abilities play distinct roles in the dynamics of number-line decision-making.

Finally, given the modest final sample size (*N* = 17), we conducted an additional bootstrap-based, Bayesian-inspired robustness analysis (see Appendix A) to evaluate the stability, uncertainty, and directional consistency of all observed correlations (H1–H3b). This post hoc analysis indicated high directional consistency across all hypotheses, with posterior distributions supporting the predicted effect directions in all cases. Although credible intervals reflected substantial uncertainty attributable to sample size, particularly for H2, posterior estimates nevertheless showed strong probabilities of direction and Bayes Factors greater than 1 across all hypotheses. Importantly, the bootstrap posterior distributions indicated that the observed associations were not driven by isolated extreme observations but reflected consistent trends across resampled datasets. The full robustness analysis is reported in Appendix A.

## 4. Discussion

In this study, we investigated how symbolic and nonsymbolic numerical abilities are integrated during number-line estimation by introducing a novel dual-format version of the NLET. The dual-NLET allowed us to examine number-line estimation behavior through the lens of the evidence accumulation paradigm by applying hierarchical drift diffusion modeling to characterize latent decision dynamics. This approach extends traditional NLET research by moving beyond outcome measures and enabling a process-level analysis of how children resolve numerical decisions.

Decision dynamics in the dual-NLET were primarily characterized by decision efficiency (drift rate) and decision threshold (boundary separation). On average, children exhibited moderate drift rates, indicating a reasonable level of efficiency in resolving competition between target and distractor marks. Boundary separation values were comparatively elevated, reflecting relatively cautious response thresholds, with children requiring a substantial amount of accumulated evidence before committing to either alternative. In cognitive terms, drift rate reflects the quality and efficiency of evidence accumulation, which can be driven by the precision of internal magnitude representations and by how effectively stimulus information is mapped onto numerical representations. Boundary separation, in contrast, reflects the decision criterion, capturing how much evidence a child requires before responding. Together, this pattern suggests a decision process combining moderate efficiency in evidence accumulation with conservative response thresholds in this preschool sample.

Accuracy in the traditional NLET was significantly associated with both accuracy and drift rate in the dual-NLET, indicating the children who showed lower estimation error in the traditional task also performed more accurately in the dual task and accumulated evidence more efficiently. These findings suggest that the two task formats relate to each other at both the outcome and process levels, consistent with prior work showing similarities across NLET variants (number-to-position and position-to-number; Siegler & Opfer, 2003; Schneider et al., 2018).

These findings directly address the first objective of the study, namely, to evaluate whether a dual-format NLET can support process-level analyses of number-line estimation. Prior work has emphasized the importance of considering both accuracy and response times in NLET performance, yet these measures have typically been analyzed separately (Ashcraft & Moore, 2012). By embedding both within a unified evidence-accumulation framework, the present approach allows latent decision components to be estimated directly. The observed associations between traditional NLET accuracy and both behavioral performance and drift rate in the dual-NLET indicate that this novel format preserves core aspects of number-line estimation while providing access to the underlying dynamics of the decision process. In this sense, our results relate to prior findings on outcome-level individual differences in children number-line estimation in the framework of the log-to-linear shift theory (Siegler & Opfer, 2003; Siegler & Booth, 2004; Opfer & Siegler, 2007) and indicate that these differences can also be investigated at the process-level using the dual-NLET for tracing underlying decision dynamics.

Beyond establishing the utility of the dual-NLET, the present findings inform theoretical accounts of how symbolic and nonsymbolic numerical abilities contribute to number-line estimation, as part of the second objective. Symbolic abilities were positively associated with decision efficiency in the dual-NLET, whereas nonsymbolic abilities were associated with decision threshold. This pattern converges with previous work showing strong links between symbolic skills and NLET accuracy (Schneider et al., 2018; Gunderson & Hildebrand, 2021), while also aligning with evidence supporting a role for nonsymbolic abilities in numerical processing (Fazio et al., 2014; Schneider et al., 2017).

Regarding the link between symbolic abilities and decision efficiency, our results are consistent with a substantial body of research demonstrating that number-line estimation is closely related to children’s symbolic numerical abilities and broader mathematical competence (Siegler & Opfer, 2003; Siegler & Booth, 2004; Opfer & Siegler, 2007; Ashcraft & Moore, 2012; Bailey et al., 2014; Jordan et al., 2013; Hornung et al., 2014; Schneider et al., 2018). In the dual-NLET, each trial requires children to map a symbolic number onto a spatial position on the number line, as in the traditional NLET, and to resolve competition between two nearby candidate locations. Within this context, we interpret decision efficiency in the dual-NLET (drift rates) not as an index of symbolic processing per se, but rather as the efficiency with which the internal numerical representation—supported by both symbolic and approximate representations—is mapped onto spatial alternatives and translated into accumulating evidence favoring the target over the distractor. Because the distance between alternatives is constant (±1 unit) across trials, variability in drift rate primarily reflects individual differences in the precision in the use of numerical representation for solving the conflict between alternatives rather than stimulus-driven differences in task difficulty. The observed association between symbolic abilities and drift rate therefore suggests that symbolic knowledge preferentially supports the efficiency of this process, while nonsymbolic abilities appear to influence complementary aspects of decision dynamics, such as response thresholds. This process-level dissociation complements traditional accounts of number-line estimation such as the log-to-linear (Siegler & Opfer, 2003; Siegler & Booth, 2004; Opfer & Siegler, 2007) by revealing how symbolic and approximate systems contribute differentially to the dynamic implementation of number-space mapping.

Regarding the association between nonsymbolic abilities—supported by the approximative system—and boundary separation, our results suggest that approximate numerical representations primarily influence the amount of evidence children require before committing to a number-line decision. In the dual-NLET, we interpret decision threshold (boundary separation) as a reflection of tolerance to uncertainty when selecting between two nearby candidate locations derived from internal magnitude representations. Children with stronger nonsymbolic abilities exhibited lower decision thresholds, indicating reduced response caution, whereas children with weaker nonsymbolic acuity required more accumulated evidence before responding. This pattern is consistent with accounts characterizing the Approximate Number System as a probabilistic system that generates inherently noisy magnitude representations (Feigenson et al., 2004; Piazza, 2010). From this perspective, individual differences in nonsymbolic acuity may shape how uncertainty is managed during estimation, with higher ANS precision supporting more confident commitments to number-line decisions.

Regarding the relation between numerical systems in the decision-making in the dual-NLET, our findings indicate that nonsymbolic abilities do not primarily enhance evidence accumulation efficiency in the dual-NLET, but instead modulate decision thresholds, revealing a complementary functional role to that of symbolic abilities in number-line decision-making. Mapping accounts propose that ANS skills constitute the foundation for the development of symbolic numerical knowledge (Dehaene & Changeux, 1993; Feigenson et al., 2004; Piazza, 2010; Szkudlarek & Brannon, 2017), whereas refinement accounts suggest that symbolic learning progressively reshapes nonsymbolic representations, placing the SNS in a predominant role and treating the ANS as complementary (Matejko & Ansari, 2016; Lyons et al., 2018; Lau et al., 2021). In this sense, our findings point toward the refinement account, in that symbolic abilities support decision efficiency in the dual-NLET, while nonsymbolic abilities complement this process by modulating children’s caution and tolerance to uncertainty.

At the same time, the present cross-sectional data do not imply that these associations are fixed across development. Rather, they provide a snapshot of system interaction at a specific developmental stage. Our results on the process-level dissociation between ANS and SNS in the dual-NLET open new possibilities for studying the developmental interaction between the numerical systems. For example, longitudinal applications could investigate whether the contributions of symbolic and nonsymbolic abilities to decision efficiency and decision threshold change over time. One possibility is that prior to formal schooling, nonsymbolic abilities may play a stronger role in shaping response criteria or early efficiency, consistent with mapping accounts. Conversely, at later developmental stages, symbolic abilities may increasingly influence both evidence accumulation and response criteria, in line with refinement accounts emphasizing top-down effects of symbolic learning on approximate representations. Such developmental shifts could be directly examined using the present dual-NLET task.

These results also relate to debates between log-to-linear and proportional accounts of number-line estimation. Proportional accounts argue that NLET performance reflects general proportional reasoning based on a single underlying mechanism (Barth & Paladino, 2011), rather than changes in numerical representations as suggested by the log-to-linear theory (Siegler & Booth, 2004). While the present study does not directly investigate representational formats, the observed dissociation between symbolic and nonsymbolic contributions poses challenges for strictly unitary interpretations. If estimation were driven by a single proportional process, one would not expect symbolic and nonsymbolic abilities to relate selectively to different latent components of the decision process. Instead, our findings support a process-level dissociation in which symbolic and approximate numerical systems make dissociable yet complementary contributions to number-line estimation behavior, with symbolic abilities specifically related to efficiency in decision dynamics, pointing toward support for the log-to-linear shift theory.

In this line of reasoning, although starting point (z) was not a focal parameter in our hypotheses, the negative mean value observed in this study invites reflection in light of the predictions of the log-to-linear shift theory. Within the diffusion modeling framework, starting point reflects an initial bias toward one of the two alternatives prior to evidence accumulation. In the present dual-NLET, a negative starting point indicates a tendency to initiate the decision process closer to the distractor mark. Because distractor positions were counterbalanced to the left and right of the target across trials, this effect reflects a general preference for the distractor rather than a direction-specific spatial tendency.

A global tendency toward the distractor cannot by itself be interpreted as evidence for logarithmic representations in the current setting. A pattern consistent with logarithmic mapping would instead require magnitude-dependent directional effects, specifically a tendency to prefer distractors located to the right of the target for small numbers (overestimation) and to the left of the target for large numbers (underestimation). The present analyses do not distinguish between such magnitude-dependent effects and more general perceptual or strategic biases and therefore do not allow this possibility to be directly evaluated.

However, the negative mean value suggests a key methodological opportunity afforded by the dual-NLET. Although the present results do not permit direct inferences about logarithmic versus linear representations, the task structure readily supports extensions aimed at probing developmental changes in numerical representations more explicitly. Within the log-to-linear framework, developmental progress is characterized by a transition from predominantly logarithmic to increasingly linear representations of numerical magnitude. Future studies could use the dual-NLET to operationalize this transition directly at the level of decision dynamics by systematically positioning the distractor at the logarithmic location of the target number while keeping the target mark at its linear position. Under such dual-NLET designs, preference for the distractor would reflect dominance of a logarithmic representation, whereas selection of the target mark would indicate linear mapping. Tracking changes in magnitude-dependent starting point bias and response accuracy across age or training could therefore provide a process-level window into representational change, allowing researchers to observe how children gradually shift from non-linear to linear numerical representations within a unified decision-making framework.

Finally, beyond methodological and theoretical implications, our results also has potential practical relevance. Distinguishing between decision efficiency and decision threshold opens the possibility of identifying different profiles of numerical difficulty in early development. Some children may struggle primarily with evidence accumulation efficiency, whereas others may exhibit elevated decision thresholds despite adequate symbolic skills. Longitudinal applications of the dual-NLET could help clarify how these profiles evolve over time and how changes in decision dynamics relate to subsequent growth in symbolic and mathematical abilities. In turn, such insights could inform future intervention studies by enabling more targeted, process-informed number-line training approaches aimed at supporting mathematical development. This perspective complements longitudinal and intervention evidence indicating systematic links between nonsymbolic and symbolic numerical development, observed both over time (Wong et al., 2016) and following structured number-line training (Morell-Ruiz et al., 2025).

### 4.1. Limitations and Future Research

Although our study initially included 26 participants, only 17 completed all measurements and were therefore available for the full set of correlational analyses. One common risk when working with small samples is that distributional assumptions required for parametric statistical tests may be violated. Accordingly, we examined the distribution of each variable and used non-parametric tests when necessary to avoid inference based on unmet assumptions. In addition, we reported effect sizes for each correlation, depending on the statistical test used. For correlations assessed with Pearson’s *r*, we followed Cohen’s (1988) conventional benchmarks. For correlations based on Spearman’s *ρ*, we used the non-parametric guidelines proposed by Dancey and Reidy (2007), which offer a more conservative interpretation of effect sizes—for example, a correlation value of *ρ* = –0.54 (between the traditional and dual-NLET accuracies) is interpreted as “moderate” according to Dancey and Reidy (2007), whereas it would be considered “large” under Cohen’s (1988) framework. This dual approach allowed us to interpret results cautiously while providing adequate context for their magnitude. Further studies with larger and more diverse samples are encouraged to confirm and extend these results.

Although the effective sample size is modest, the present study represents the first application of two-choice diffusion modeling to number-line estimation, enabled by the novel dual-NLET design. Previous diffusion-based work in this domain has relied on spatially continuous models in adult samples, which are grounded in different principles and are explicitly not recommended for developmental data (Ratcliff & McKoon, 2020, 2024). By contrast, the dual-NLET transforms estimation into a binary decision format, allowing number-line performance to be examined within a discrete evidence-accumulation framework. While two-choice diffusion models have previously been applied to numerical two-alternative forced-choice tasks in both children (Szardenings et al., 2018; Spliethoff et al., 2022) and adults (Park & Starns, 2015), this is, to our knowledge, the first study to integrate such a framework with the NLET.

Given the methodological complexity of combining individual testing in preschoolers with multi-task assessment and hierarchical diffusion modeling, some reduction in the effective sample size was inevitable. Importantly, the aims of the study were to test whether the dual-NLET can be used as a process-level extension of the traditional task and to examine whether symbolic and nonsymbolic numerical abilities map onto distinct components of decision dynamics during number-line estimation. To address uncertainty associated with sample size, we complemented frequentist analyses with bootstrap-based posterior approximations, which indicated consistent directional effects across hypotheses (see Appendix A). Accordingly, the present findings should be interpreted as an initial demonstration of the dual-NLET as a process-level extension of the traditional task and functional dissociation between symbolic and nonsymbolic contributions within the decision dynamics of number-line estimation. Replication in larger samples will help further establish the robustness and generalizability of these process-level effects.

Finally, future developmental research could integrate the dual-NLET with longitudinal or cohort-sequential designs to investigate how the dynamics of numerical decision-making unfold over time. Gunderson and Hildebrand (2021) demonstrated that number-line estimation remains consistently related to multiple facets of numerical competence across development, but their approach did not examine the mechanisms underlying estimation decisions. Building on their developmental evidence and our process-level results, future studies could explore whether the distinct contributions of symbolic and nonsymbolic abilities to decision efficiency and decision caution evolve with age. For instance, one possibility is that decision efficiency increases more rapidly as symbolic representations become more precise with schooling, whereas decision caution—linked to nonsymbolic intuition—may gradually decrease as children rely less on approximate information. Such hypotheses remain open for empirical testing, and developmental applications of the dual-NLET could offer valuable insights into how numerical systems shape decision-making across childhood.

### 4.2. Contributions of the Study

This study makes methodological, theoretical and practical contributions to the investigation of number-line estimation and early numerical decision-making. Methodologically, we introduce the dual-NLET as a novel two-alternative forced-choice adaptation of the NLET that enables the application of two-choice diffusion modeling to number-line estimation. By integrating accuracy and response times within a unified evidence-accumulation framework, this approach moves beyond traditional outcome-based analyses of accuracy and response times and provides direct access to core components of decision dynamics, including decision efficiency and decision threshold. To our knowledge, this is the first application of hierarchical drift diffusion modeling to number-line estimation in children and the first implementation of two-choice diffusion modeling in the NLET context, offering a new process-oriented framework that bridges developmental numerical cognition and decision-making research, enabling examination of how numerical decisions unfold in real time. The dual-NLET thus provides a complementary tool for studying number-line estimation, linking numerical representation and decision dynamics within a single developmental paradigm.

Theoretically, our findings reveal a functional dissociation in the decision-making dynamics of estimation, with symbolic abilities primarily supporting decision efficiency and nonsymbolic abilities modulating decision threshold. This pattern supports a process-level dissociation in which symbolic and approximate systems make dissociable yet complementary contributions to numerical decision-making. By grounding these conclusions in decision dynamics rather than accuracy and response times alone, the present study adds nuance to existing accounts of numerical estimation and highlights the value of process-level approaches for advancing developmental numerical cognition research.

From a practical perspective, distinguishing between decision efficiency and decision threshold provides a principled way to characterize individual differences in early numerical estimation beyond accuracy alone. This process-level separation opens the possibility of identifying distinct profiles of numerical difficulty, distinguishing children whose challenges primarily reflect difficulties with evidence accumulation from those whose performance is driven by elevated response caution despite adequate symbolic skills. Such differentiation has potential implications for early assessment and educational support, as it suggests that children may benefit from different forms of number-line intervention depending on their underlying decision dynamics.

In summary, this study contributes to the field by introducing the dual-NLET as a methodological extension of the traditional number-line estimation task that enables process-level modeling of numerical decisions, and by providing initial theoretical evidence that symbolic and nonsymbolic abilities make dissociable contributions to decision dynamics. In addition, by separating decision efficiency from decision threshold, the present framework offers a practical perspective on individual differences in early numerical estimation, highlighting the potential for identifying distinct decision profiles and informing more targeted, process-sensitive educational approaches. Together, these methodological, theoretical, and practical contributions suggest a novel framework for studying number-line estimation beyond outcome-based measures and for characterizing how symbolic and approximate systems jointly shape children’s numerical decision-making.

## 5. Conclusions

The present study examined number-line estimation from a process-oriented perspective by applying an evidence accumulation framework to a novel two-alternative forced-choice version of the Number Line Estimation Task (dual-NLET). By combining this task with hierarchical drift diffusion modeling, we aimed to explore how symbolic and nonsymbolic numerical abilities relate to different aspects of decision-making during numerical estimation.

The results suggest that symbolic and nonsymbolic numerical abilities are both involved in number-line estimation but may be associated with different components of the decision process. Symbolic numerical abilities were related to decision efficiency, as reflected in higher drift rates, indicating more effective evidence accumulation toward the correct alternative. In contrast, nonsymbolic numerical abilities were related to decision threshold, as reflected in boundary separation, suggesting differences in response caution during estimation. These findings point to a process-level dissociation in which symbolic and nonsymbolic systems make complementary contributions to numerical decision-making, with symbolic abilities preferentially supporting evidence accumulation efficiency and nonsymbolic abilities shaping response thresholds.

From a methodological perspective, the dual-NLET provides an alternative format for studying number-line estimation that allows the use of two-choice diffusion models. The observed associations between performance in the traditional NLET and both accuracy and decision efficiency in the dual-NLET indicate that the two task formats are related. This suggests that the dual-NLET may be useful as a complementary tool for examining the cognitive processes underlying number-line estimation.

Overall, the present findings illustrate how process-level analyses can add nuance to existing accounts of numerical estimation by distinguishing between decision efficiency and decision threshold. Beyond theoretical insight, this separation provides a framework for characterizing individual differences in early numerical estimation, highlighting the potential to identify distinct decision profiles that may be relevant for educational assessment and targeted number-line interventions. While the current sample size is modest, the results point to the value of applying two-choice diffusion modeling to the study of number-line estimation using the dual-NLET, both for advancing theory and for informing process-sensitive approaches to early mathematical learning in future research.

## Figures and Tables

**Table 1:** Mean and Standard Deviation for the measures included in the study (*N* = 17) together with Shapiro–Wilk test of normality.

Measure	*M*	*SD*	*W*	*p*
Accuracy in the traditional NLET (PAE) ^1^	11.44	6.6	0.88	<.05
Accuracy in the dual-NLET (% correct)	67.65	10.42	0.86	<.05
Decision efficiency in the dual-NLET (drift rate)	0.36	0.05	0.93	.23
Decision threshold in the dual-NLET (boundary separation)	2.9	0.51	0.94	.33
Symbolic numerical abilities	32	5.9	0.96	.64
Nonsymbolic numerical abilities	32.88	4.33	0.95	.49

^1^ PAE = Percentage Absolute Error. Shapiro–Wilk statistics correspond to tests of normality.

**Figure A1:**
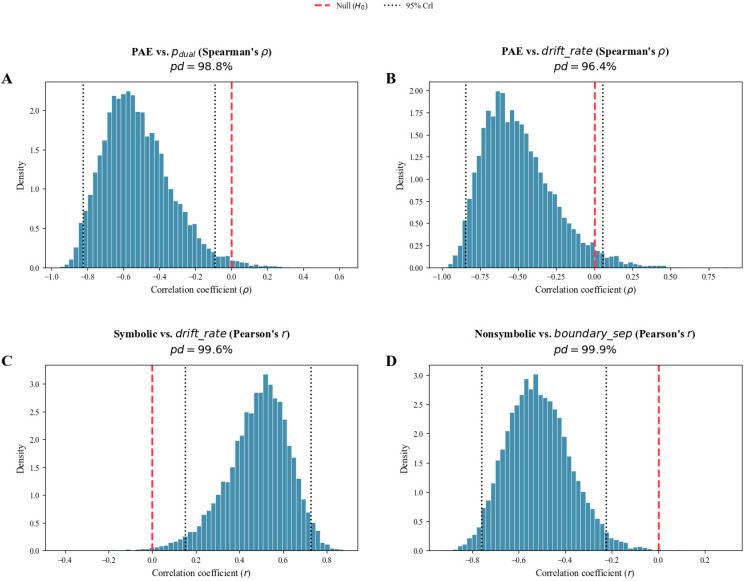
Bayesian posterior distributions for the four core hypotheses. Panels (**A**,**B**) show Spearman’s rank correlations (*ρ*), while panels (**C**,**D**) show Pearson’s correlations (*r*). The light blue histograms represent the posterior distributions generated via 10,000 bootstrap iterations. The red dashed line indicates the null hypothesis (no correlation), and the black dotted lines denote the 95% Credible Intervals (*CrI*). *P* represents the Probability of Direction (*pd*), indicating the certainty that the observed effect exists in the predicted direction.

## Data Availability

The raw data supporting the conclusions of this article will be made available by the authors on request.

## Appendix A. Robustness Check: Bootstrap-Based Bayesian-Inspired Correlation Analysis

The original sample in the current study comprised 26 participants but only 17 completed all measurements required for the full set of correlational analyses, individual observations may have relatively strong leverage on the estimated effects. In this context, conventional significance testing alone may provide an incomplete picture of uncertainty and effect stability.

To address this limitation, this robustness check uses bootstrap resampling to approximate posterior distributions of the correlation coefficients. From these empirical distributions, three complementary quantities are derived: Probability of Direction (*pd*), 95% Credible Interval (*CrI*), and Bayes Factor (${BF}_{10}$). The Probability of Direction reflects the posterior probability that an effect lies in the predicted direction, ranging from 50% (no directional preference) to 100% (complete directional consistency). The 95% Credible Interval represents the range within which the correlation coefficient falls with 95% posterior probability, providing a direct measure of parameter uncertainty. The Bayes Factor quantifies the relative evidence in favor of the experimental hypothesis (non-zero correlation in the predicted direction) compared to the null hypothesis (exactly zero correlation), with values above 1 indicating support for the experimental prediction.

Together, these metrics address directionality, uncertainty, and strength of evidence.

For each hypothesis in the study, we first computed the observed correlation coefficient using Spearman’s *ρ* for H1 and H2, and Pearson’s *r* for H3a and H3b, consistent with the analyses included in the Result section in the manuscript. Uncertainty was then characterized via 10,000 bootstrap resamples, from which posterior approximations were obtained and used to estimate the 95% *CrI* and *pd*.

To compute Bayes Factors (${BF}_{10}$), we evaluated the likelihood of the observed correlation under different possible true correlations across the interval [−1, 1], using a uniform prior that assigns equal plausibility to all correlation values and therefore minimizes prior assumptions.

Integrating this likelihood yielded the marginal likelihood for the alternative hypothesis, which was then divided by the likelihood under the null hypothesis (correlation = 0). Because this analytical step is formally derived for Pearson correlations, Spearman’s *ρ* was treated as an effect-size approximation for this post hoc robustness analysis.

This procedure yields an internally consistent framework for interpreting the observed effects in the context of small-sample uncertainty. Table 1 summarizes the resulting robustness metrics for all four hypotheses. Probability of Direction values are high across hypotheses (all above 96%), indicating consistent alignment with the predicted directions. Credible intervals indicate moderate uncertainty, with H2 in particular including zero, reflecting limited precision in effect magnitude while preserving strong directional consistency. Bayes Factors are all above 1 and fall in the weak-to-moderate range, indicating consistent, albeit modest, evidence in favor of the experimental predictions.

Figure A1 provides a visual diagnostic of these outcomes. Panels A–D of Figure A1 correspond to H1, H2, H3a, and H3b, respectively The histograms depict the bootstrap-derived posterior distributions of the correlation coefficients, while the red dashed line marks the null hypothesis (correlation = 0). Black dotted lines indicate the 95% Credible Intervals. The relative displacement of each distribution from zero illustrates the direction of the effects, and the spread of each distribution reflects remaining uncertainty. Consistent with Table A1, H2 (panel B) shows broader dispersion and a credible interval overlapping zero, whereas the remaining hypotheses display posterior mass more clearly separated from the null.

Importantly, the posterior distributions do not indicate that the observed effects are driven by isolated extreme observations; instead, they reflect consistent trends across resampled datasets.

**Table A1:** Bootstrap-based Bayesian-inspired robustness metrics for the four correlation hypotheses (*N* = 17).

Hypothesis (H)	Relation Between Variables (Test)	Prob. of Direction (*pd*)	95% *CrI*	$\mathrm{Bayes}\;\mathrm{Factor}\;({BF}_{10})$
H1	PAE vs. p_dual (Spearman)	98.9%	[−0.823, −0.096]	3.108
H2	PAE vs. drift_rate (Spearman)	96.6%	[−0.846, 0.046]	2.597
H3a	Symbolic vs. drift_rate (Pearson)	99.4%	[0.151, 0.731]	1.874
H3b	Nonsymbolic vs. boundary_sep (Pearson)	99.8%	[−0.762, −0.212]	2.224

Note: Hypothesis (H) refers to the specific experimental prediction. Probability of Direction (pd) represents the posterior probability that the correlation is in the predicted direction (negative for H1, H2, and H3b; positive for H3a), providing a continuous measure of directional consistency. CrI = 95% Credible Interval, estimated via bootstrap approximation given the small sample size (*N* = 17). BF10 denotes the Bayes Factor in favor of the experimental hypothesis relative to the null.

In summary, this post hoc analysis provides complementary robustness evidence for the main findings using a bootstrap-based Bayesian-inspired framework tailored to the constraints of the available data (26 originally recruited participants, with 17 completing all relevant measures). The analysis indicates that all four hypothesized relationships are directionally consistent with theoretical predictions, with Bayes Factors consistently above 1, weak-to-moderate relative evidence, and varying degrees of uncertainty in effect size.

While the modest sample size necessarily limits inferential strength, these results support the overall pattern observed in the frequentist analyses and suggest that the reported associations are not artifacts of single observations. At the same time, the wider credible interval for H2 highlights the need for cautious interpretation and underscores the value of future studies with larger samples. Taken together, the combined frequentist and bootstrap-based Bayesian-inspired results point to coherent and stable directional effects, while transparently acknowledging remaining uncertainty.
